# Investigating the concordance of Gene Ontology terms reveals the intra- and inter-platform reproducibility of enrichment analysis

**DOI:** 10.1186/1471-2105-14-143

**Published:** 2013-04-29

**Authors:** Lifang Zhang, Juan Zhang, Gang Yang, Di Wu, Lina Jiang, Zhining Wen, Menglong Li

**Affiliations:** 1 College of Chemistry, Sichuan University, Chengdu, 610064, People's Republic of China; 2 State Key Laboratory of Chemo/Biosensing and Chemometrics, Hunan University, Changsha, 410082, People's Republic of China

**Keywords:** DNA microarray, Intra-/inter-platform comparison, Gene Ontology enrichment, Microarray quality control (MAQC)

## Abstract

**Background:**

Reliability and Reproducibility of differentially expressed genes (DEGs) are essential for the biological interpretation of microarray data. The microarray quality control (MAQC) project launched by US Food and Drug Administration (FDA) elucidated that the lists of DEGs generated by intra- and inter-platform comparisons can reach a high level of concordance, which mainly depended on the statistical criteria used for ranking and selecting DEGs. Generally, it will produce reproducible lists of DEGs when combining fold change ranking with a non-stringent p-value cutoff. For further interpretation of the gene expression data, statistical methods of gene enrichment analysis provide powerful tools for associating the DEGs with prior biological knowledge, e.g. Gene Ontology (GO) terms and pathways, and are widely used in genome-wide research. Although the DEG lists generated from the same compared conditions proved to be reliable, the reproducible enrichment results are still crucial to the discovery of the underlying molecular mechanism differentiating the two conditions. Therefore, it is important to know whether the enrichment results are still reproducible, when using the lists of DEGs generated by different statistic criteria from inter-laboratory and cross-platform comparisons. In our study, we used the MAQC data sets for systematically accessing the intra- and inter-platform concordance of GO terms enriched by Gene Set Enrichment Analysis (GSEA) and LRpath.

**Results:**

In intra-platform comparisons, the overlapped percentage of enriched GO terms was as high as ~80% when the inputted lists of DEGs were generated by fold change ranking and Significance Analysis of Microarrays (SAM), whereas the percentages decreased about 20% when generating the lists of DEGs by using fold change ranking and *t*-test, or by using SAM and *t*-test. Similar results were found in inter-platform comparisons.

**Conclusions:**

Our results demonstrated that the lists of DEGs in a high level of concordance can ensure the high concordance of enrichment results. Importantly, based on the lists of DEGs generated by a straightforward method of combining fold change ranking with a non-stringent p-value cutoff, enrichment analysis will produce reproducible enriched GO terms for the biological interpretation.

## Background

Over the last decade, DNA microarray technology has reached a rapid development and found wide application in many areas of biology and medical science. One of its important applications is to identify differentially expressed genes (DEGs) across groups of samples or distinct biological conditions of interest [1,2]. Biological interpretation of microarray data requires reliable and reproducible lists of DEGs. The microarray quality control (MAQC) project launched by US Food and Drug Administration (FDA) elucidated that the lists of DEGs generated by intra- and inter-platform comparisons reached a high level of concordance, which largely depended on the statistical criteria used for ranking and selecting DEGs [3,4]. For the further biological interpretation, statistical methods of gene enrichment analysis provide powerful tools for associating the DEGs with prior biological knowledge, e.g. Gene Ontology (GO) terms and signaling pathways. The enrichment analysis mainly used prior knowledge, e.g. GO categories [5,6] or Kyoto Encyclopedia of Genes and Genomes (KEGG) pathways [7,8], to investigate whether the predefined gene sets showed significantly phenotypic differences between two biological states.

Many methods for enrichment analysis were developed to discover the biological meaning of DEGs. Mootha et al. firstly proposed an earlier version of Gene Set Enrichment Analysis (GSEA), which used an equal weighted version of Kolmogorov-Smirnow statistic for gene sets enrichment without considering the correlation between genes and the phenotype [9]. Subramanian et al. extended this procedure in 2005 and successfully used it for analyzing molecular profiling data [10]. Kim and Volsky carried out a parametric analysis of gene set enrichment (PAGE) to the improved GSEA and identified more statistically significant gene sets. PAGE used less computational effort than GSEA because it used normal distribution for statistical inference [11]. Oron et al. improved GSEA by using a linear regression diagnostic technique and discovered a vital factor to the influence of gene expression from acute lymphoblastic leukemia datasets [12]. Ji et al. proposed a new method FDR-FET to improve the sensitivity and selectivity of GSEA [13]. Kim et al. used z-statistics and permutation test to identify significantly enriched gene sets [14]. In addition, other statistical methods including significance analysis of function and expression (SAFE) [15], BayGO [16], ProbCD [17], EasyGO [18], ProfCom [19], GlobalANCOVA [20], GOEAST [21] and LRpath [22] were also developed for enrichment analysis.

Based on the methods mentioned above, researchers can subsequently reveal the pathological mechanism from the microarray data sets. Xu et al. enriched two gene sets associated with the glycolytic-related pathway from the microarray data of prostate non-recurrent patients. This pathway was considered as a candidate negative modulator of AKT1-induced proliferation [23]. De Windt et al. used GSEA to analyze Niemann-pick type C (NPC) disease and discovered 27 up-regulated and 33 down-regulated pathways. These affected pathways were provided as targets for subsequent drug discovery project [24]. In breast cancer research, Murohashi et al. found that the genes composed in CD24/^low^/CD44^+^ cell populations were fallen into the significantly enriched gene sets, which were associated with the pathways of transforming growth factor-ß, tumor necrosis factor, and interferon response. The signaling pathways enriched by GSEA were suggested to identify molecular targets and biomarkers for Tumour-initiating-like cells [25].

However, when mapping the DEGs to the predefined gene sets, any difference between two DEG lists may cause different outputs of the enrichment analysis. For the same compared conditions, the reproducible enrichment results are still crucial to the discovery of the underlying molecular mechanism differentiating the two conditions. Therefore, it is important to know whether the enrichment results are still reproducible, when using the lists of DEGs generated by different statistic criteria from different commercial microarray platforms. As a part of the MAQC project, Guo et al. investigated the intra-laboratory overlap of enriched KEGG pathways and GO terms with a rat toxicogenomics dataset and revealed that, compared to the p-value ranking, the use of fold change ranking (with p < 0.05 cutoff) for DEG selection showed more consistency in enrichment analysis [26]. In the previous study by Manoli et al. [27], the concordance of pathways enriched by Fisher’s exact test, global test and GESA were investigated based on the microarray data from Affymetrix microarray platform and the DEGs generated by significance analysis of microarrays (SAM) and mixed model analysis (MMA). The pathways found by Fisher’s exact test and global test showed more concordant than those by GSEA in all conditions. In the current study, the microarray data were collected from the large data sets provided by MAQC project [3,4], which included three major microarray platforms: Affymetrix (AFX), Agilent Technologies (AG1) and Illumina (ILM) and the lists of DEGs for enrichment analysis were generated by using three statistical criteria: fold change ranking with a non-stringent p-value cut-off which was calculated by *t*-test, significance analysis of microarrays (SAM) [28] and *t*-test. Finally, we systematically investigated the intra- and inter-platform concordance of GO terms enriched by two common methods of enrichment analysis, namely gene set enrichment analysis (GSEA) [10] and LRpath [22]. The results showed that, based on the DEG lists generated by SAM and FC, the levels of intra- and inter-platform concordance of GO terms were generally high and can satisfy the further biological interpretation.

## Results

**Figure 1:**
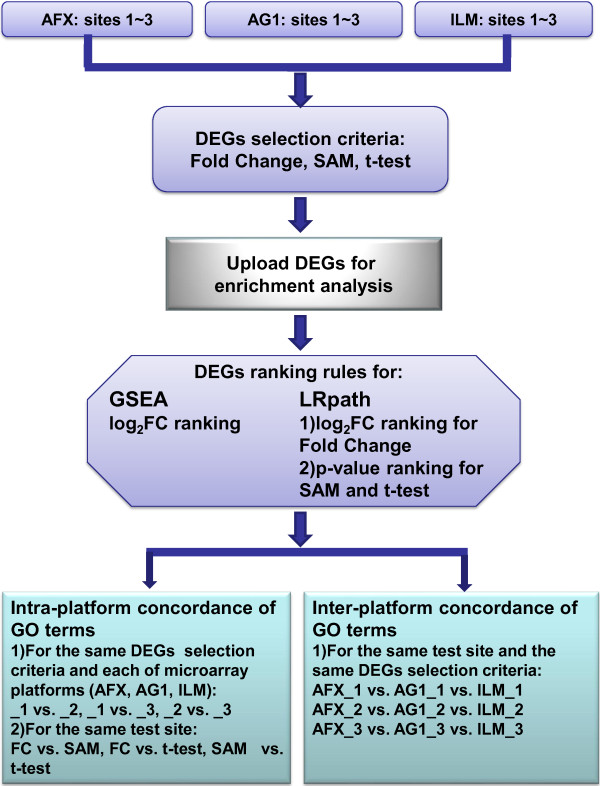
**Schematic overview of the concordance analysis of enriched gene ontology terms.** The _1, _2 and _3 suffixes refer to test site location. DEGs refer to differentially expressed genes.

In this study, we systematically investigated whether the results of enrichment analysis were still reproducible when the inputted lists of DEGs were generated by three statistical methods from different commercial microarray platforms. The GO terms were enriched by using GSEA and LRpath with the criteria of FDR < 0.25. Then, the intra- and inter-platform concordance of these terms was analyzed (Figure 1) and detailed results were shown below.

### Intra-platform concordance of enrichment results

**Figure 2:**
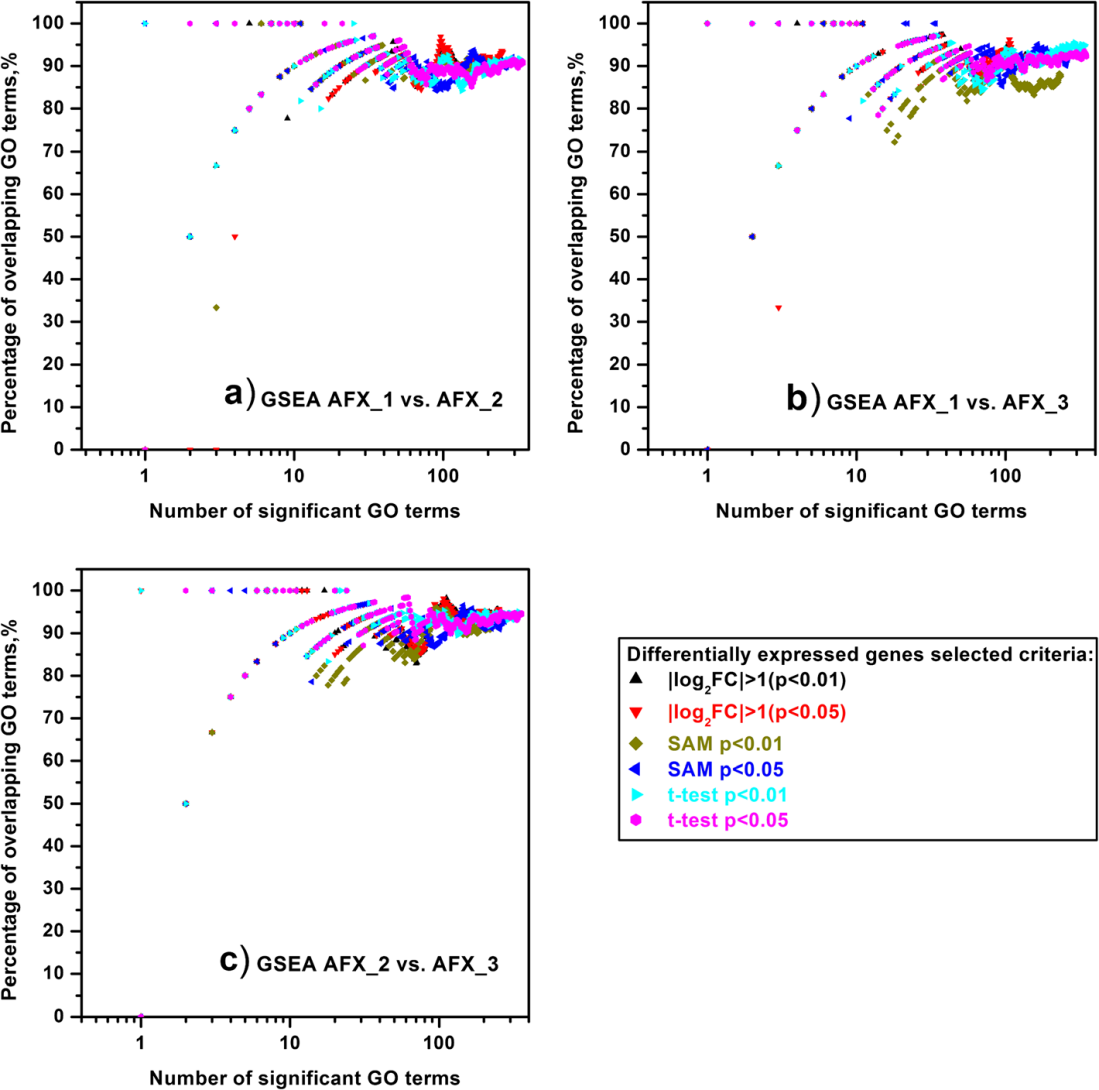
**Intra**-**platform concordance of significant GO terms enriched by GSEA among three test sites of AFX.** (**a**) AFX_1 versus AFX_2; (**b**) AFX_1 versus AFX_3; (**c**) AFX_2 versus AFX_3. The scatter plots showed the percentage of overlapping GO terms which were enriched by GSEA and derived from two test sites. Each color and each type of points represented the different gene lists that selected by different statistical methods and different cutoff. The x-axis represents the number of enriched GO terms selected as significance, and y-axis is the percentage (%) of GO terms common to the two AFX test sites enrichment results. The _1, _2 and _3 suffixes refer to test site locations.

**Figure 3:**
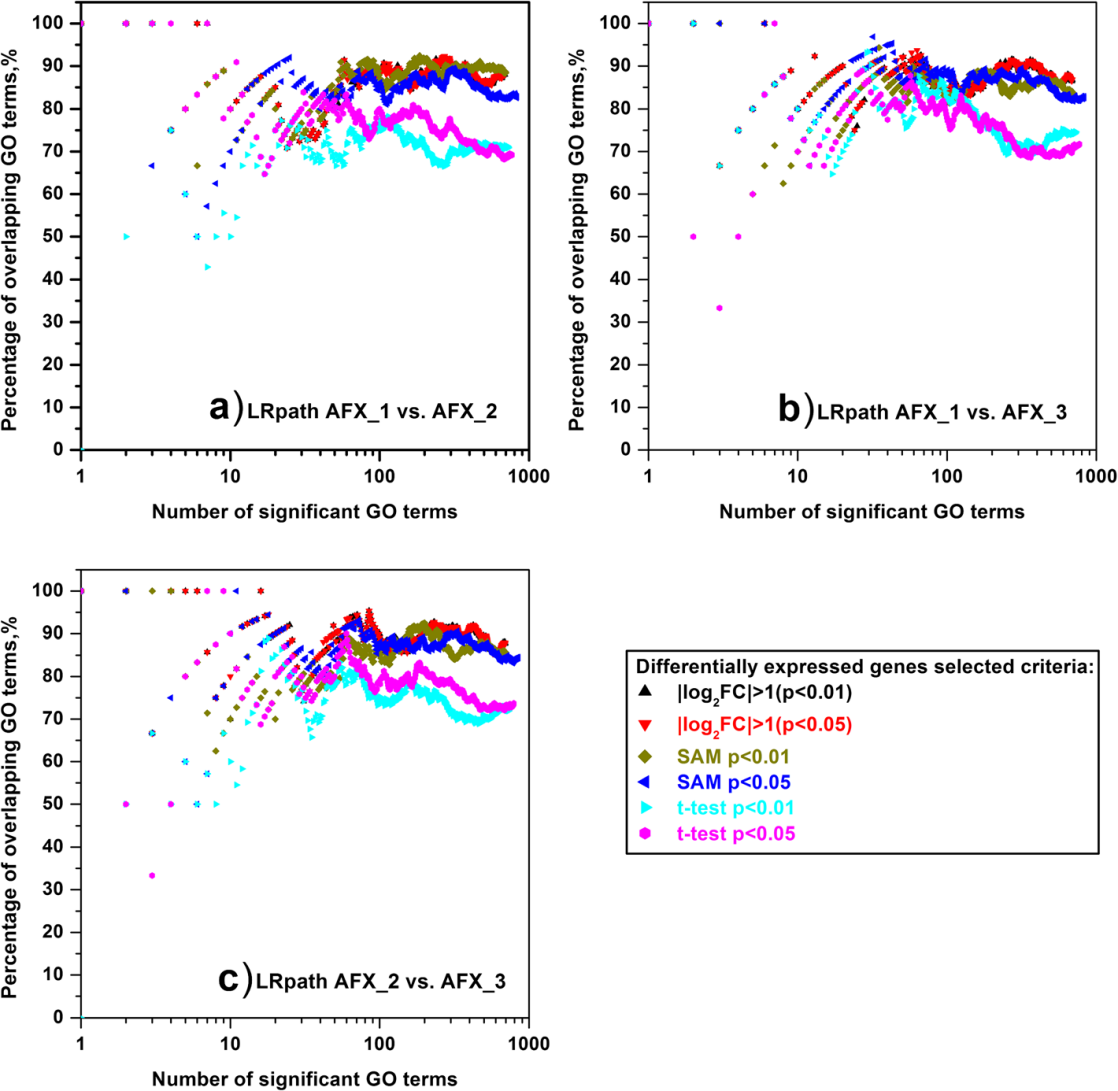
**Intra**-**platform concordance of significant GO terms enriched by LRpath among three test sites of AFX.** (**a**) AFX_1 versus AFX_2; (**b**) AFX_1 versus AFX_3; (**c**) AFX_2 versus AFX_3. The scatter plots showed the percentage of overlapping GO terms which were enriched by LRpath and derived from two test sites. See notes under Figure 2 for more information.

For the intra-platform comparison, we inspected the concordance of significant GO terms enriched by GSEA and LRpath when 1) the inputted lists of DEGs were generated from different test sites by using the same statistic criteria, and 2) the inputted lists of DEGs were generated by using different statistic criteria from the same test sites. Based on the expression data generated from Affymetrix microarray platform, the inter-site comparisons were conducted and the percentages of overlapping significant GO terms enriched by GSEA and LRpath were shown in Figure 2 and Figure 3, respectively. When selecting the top n GO terms (n ≥ 10), it can be seen from Figure 2 that all the percentages of overlapping GO terms were as high as ~90%, which indicates high inter-site concordance among the GO terms enriched by GSEA. For the inter-site concordance of GO terms enriched by LRpath (Figure 3), the percentages of overlapping GO terms were still around 87% for two DEG selection methods, fold change ranking with a non-stringent p-value cut-off and SAM, when all the GO terms meeting the FDR < 0.25 criterion were selected. In addition, for the DEG selection method of *t*-test, the overlapped percentages were about 19% lower than those showed in Figure 3 (a drop from ~88% to ~69%), suggesting that the inter-site concordance of GO terms for *t*-test was less reproducible than those for SAM and fold change ranking.

The inter-site concordance comparisons were also conducted for AG1 and ILM (Additional file 1: Figures S1-S4). Similar to the analysis results for AFX, based on the microarray data from AG1, the concordance of GO terms enriched by GSEA for all the DEG selection criteria was generally high (~90%) when all the GO terms meeting FDR < 0.25 criterion were selected (Additional file 1: Figure S1) and a significant drop of percentages (a drop from ~84% to ~63%) was also seen for *t*-test method when the GO terms were enriched by LRpath (Additional file 1: Figure S2). Note that there was an obvious drop of percentages for SAM with a cut-off of p < 0.01, because the number of DEGs selected by SAM with p < 0.01 was less than half of those selected by other DEG selection methods (Table 1). As to the results for ILM, the percentages of overlapping GO terms for all DEG selection methods but SAM were higher than ~89%, when the GO terms were enriched by GSEA (Additional file 1: Figure S3). For SAM with p < 0.01, the percentages of overlapping GO terms were as high as ~89% when comparing the test sites 1 with 3 (Additional file 1: Figure S3b), whereas the percentages dropped to ~80% when comparing the test sites 1 with 2 (Additional file 1: Figure S3a) and ~76% when comparing the test sites 2 with 3 (Additional file 1: Figure S3c). The main reason for the decrease in percentages was the reduction in the number of DEGs selected from test site 2, which was only 3,059 for test site 2 and were 5,192 and 6,996 for test sites 1 and 3, respectively (Table 1). It suggested that inter-site concordance of GO terms were also impacted by the number of selected DEGs. In addition, for SAM with p < 0.05, the percentage was dropped to ~85% when comparing the test sites 2 with 3 (Additional file 1: Figure S3c), which was ~5% lower than those when comparing the test sites 1 with 2 and the test sites 1 with 3. When the GO terms were enriched by LRpath, only the percentages of overlapping GO terms for the DEG selection method of fold change ranking with a non-stringent p-value cut-off were higher than ~80%. The percentages of overlapping GO terms for the rest DEG selection methods varied from ~55% to ~73% (Additional file 1: Figure S4).

**Table 1:** **Number of DEGs selected by FC**, **SAM and *t***-**test and different cutoff**

	**AFX_1**	**AFX_2**	**AFX_3**	**AG1_1**	**AG1_2**	**AG1_3**	**ILM_1**	**ILM_2**	**ILM_3**
|log_2_FC| > 1 (p < 0.01)	4442	4236	4546	5405	5601	5460	3677	3355	3281
|log_2_FC| > 1 (p < 0.05)	4444	4237	4548	5539	5736	5554	3677	3361	3281
SAM (P < 0.01)	4665	4269	3726	2623	2658	2840	5192	3059	6996
SAM (P < 0.05)	6682	6485	6287	5495	5553	5692	6634	5657	8071
*t*-test (p < 0.01)	9246	9322	9528	7447	8209	8180	7757	6497	7036
*t*-test (p < 0.05)	9920	9982	10187	8302	8925	9018	8649	7642	8021

There are three microarray platforms, and three test sites for each platform. For the convenient of intra- and inter-platform comparison, we directly focused on the expression of 12,091 common probes based on matching of One Probe-to-One Gene List summarized by MAQC project (http://www.nature.com/nbt/journal/v24/n9/extref/nbt1239-S5.txt). DEGs were selected by three different statistical methods for enrichment analysis, namely fold change ranking with a non-stringent p-value cut-off, SAM and *t*-test. Different cutoffs were applied in this research.

**Figure 4:**
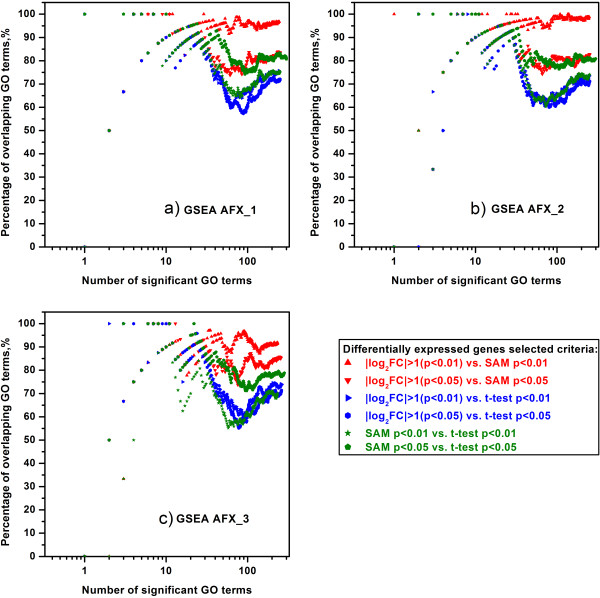
**The concordance of significant GO terms enriched by GSEA within the same test site of AFX.** The comparisons of significant GO terms enriched by GSEA were conducted within **a**) AFX_1; **b**) AFX_2; **c**)AFX_3. Two DEG lists generated by fold change ranking, two DEG lists generated by SAM and two DEG lists generated by *t*-test were inputted in GSEA to enrich significant GO terms (FDR < 0.25). And then the concordance of two significant GO terms lists derived from two DEGs selected methods was compared on the same p-value cutoff. The red markers reflect the percentages of overlapping GO terms by using fold change ranking and SAM for generating DEG lists. The blue markers reflect the percentages of overlapping GO terms by using fold change ranking and *t*-test for generating DEG lists. The olive markers reflect the percentages of overlapping GO terms by using SAM and *t*-test for generating DEG lists. The _1, _2 and _3 suffixes refer to test site location.

**Figure 5:**
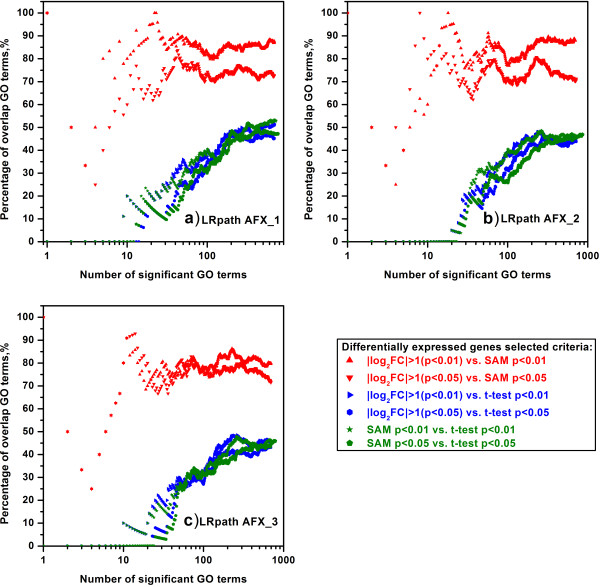
**The concordance of significant GO terms enriched by LRpath within the same test site of AFX.** The comparisons of significant GO terms enriched by LRpath were conducted within **a**) AFX_1; **b**) AFX_2; **c**)AFX_3. The same test site of the same platform enrichment which used the gene lists derived from different statistical methods. Two DEGs lists generated by fold change ranking and two DEGs lists generated by SAM and two DEGs lists generated by *t*-test were input in LRpath to get significant GO terms (FDR < 0.25). See notes under Figure 4 for more information. The concordance of two significant GO terms lists derived from two DEGs selected methods was compared on the same p-value cutoff.

In order to demonstrate the difference among the lists of GO terms created by GSEA and LRpath with the inputted DEGs generated by different DEG selection criteria, we compared the percentages of overlapping GO terms for each microarray platform at each test site. For a certain test site, the comparisons of three DEG selection methods, namely fold change ranking with a non-stringent p-value cut-off versus SAM, fold change ranking with a non-stringent p-value cut-off versus *t*-test and *t*-test versus SAM, were conducted. Figures 3 and 4 showed the concordance of GO terms enriched by GSEA and LRpath, respectively, for the Affymetrix microarray platform at three test sites. When the GO terms enriched by GSEA, most of the percentages of overlapping GO terms for the comparison of fold change ranking with a non-stringent p-value cut-off and SAM were greater than ~82%, which were the highest percentages among the comparisons of DEG selection methods (Figure 4). Especially for the comparison of fold change (|log_2_FC| > 1 (p < 0.01)) and SAM (p < 0.01), the percentages were as high as ~98% when all GO terms meeting FDR < 0.25 criterion were selected. When comparing the fold change ranking with *t*-test and SAM with *t*-test, the percentages of overlapping GO terms varied from ~70% to ~81%, which were about 20% lower than those for comparing fold change ranking with SAM. However, for the GO terms enriched by LRpath, Figure 5 showed the more obvious difference between the percentages for comparing fold change ranking with SAM and those for the comparisons of fold change ranking with *t*-test and SAM with *t*-test. The percentages of overlapping GO terms for comparing the fold change ranking with SAM were higher than ~71%, whereas the percentages for the comparisons of fold change ranking versus *t*-test and SAM versus *t*-test were lower than ~52% when all GO terms meeting FDR < 0.25 criterion were selected.

The results of the comparisons among difference DEG selection criteria for AG1 and ILM were shown in Additional file 1: Figures S5-S8. For AG1, when the GO terms enriched by GSEA and LRpath, the percentages of overlapping GO terms for comparing the fold change ranking (p < 0.05) with SAM (p < 0.05) were always higher than ~77%, whereas the percentages of overlapping GO terms for the rest comparisons varied from ~62% to ~92% (Additional file 1: Figure S5 and S6). Similar results can be seen for ILM (Additional file 1: Figure S7 and S8). When all GO terms meeting FDR < 0.25 criterion were selected, the variation range of the percentages for the comparisons among three DEG selection criteria became wider than those showed in Additional file 1: Figures S5 and S6.

### Inter-platform concordance of enrichment results

**Figure 6:**
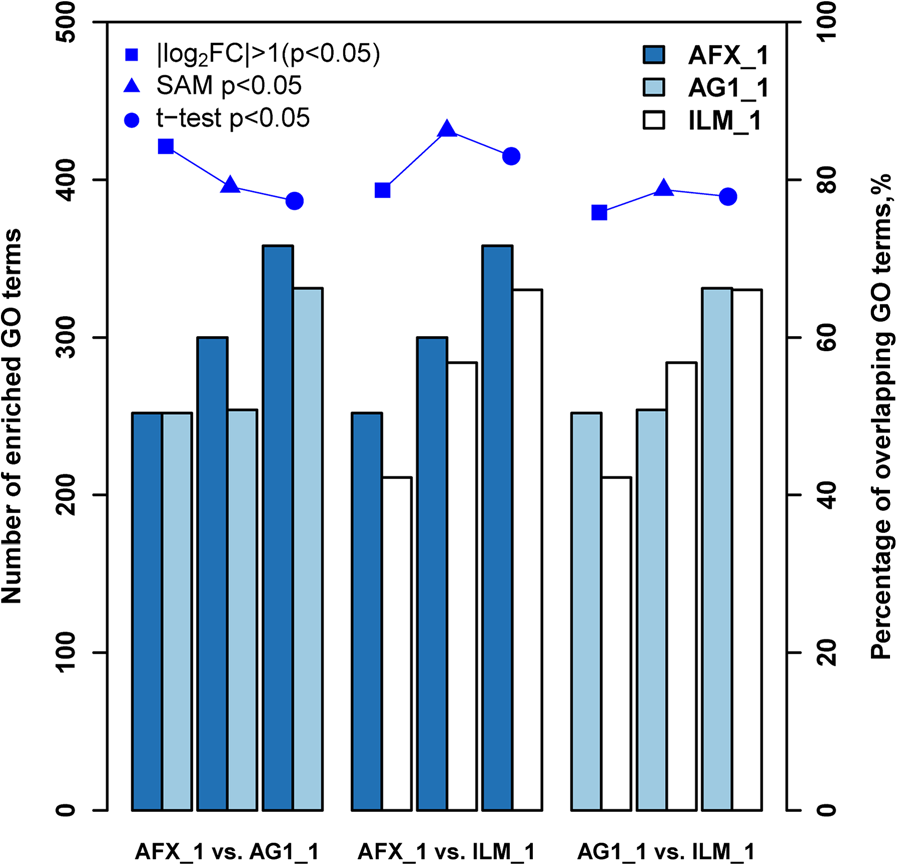
**Inter**-**platform concordance of significant GO terms enriched by GSEA.** The left y-axis represents the number of enriched GO terms by using GSEA for each of the platforms, and the right y-axis is the percentage (%) of overlapping GO terms. The number of overlapping GO terms of each platform are presented in a series of bars which correspond to the left scale. Scatter plots represent the percentages of overlapping GO terms of two platforms which correspond to the right scale. The blue, lightblue and white barplots represent the number of significantly enriched GO terms (FDR < 0.25) of AFX_1, AG1_1 and ILM_1, respectively. Different markers represent different DEG selection methods and their cutoff. The _1 suffixe refers to test site.

**Figure 7:**
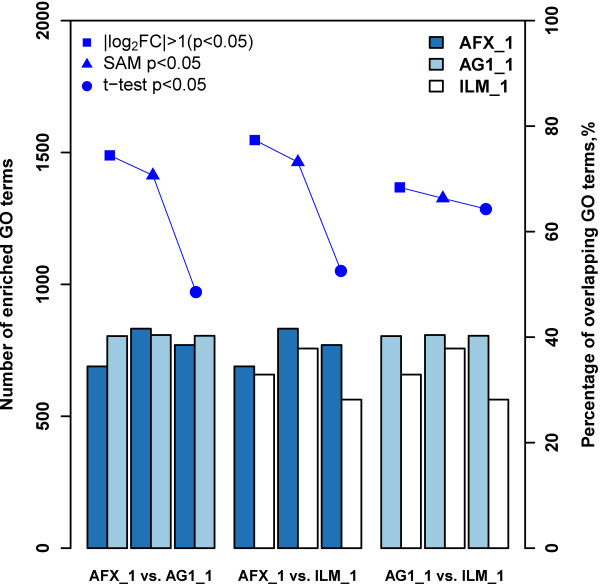
**Inter**-**platform concordance of significant GO terms enriched by LRpath.** The left y-axis represents the number of enriched GO terms by using LRpath for each of the platforms, and the right y-axis is the percentage (%) of overlapping GO terms. See notes under Figure 6 for more information. We can clearly see a drop of the percentages for DEG selection criteria *t*-test.

With regard to the analysis of inter-platform concordance, we inspected the percentages of overlapping GO terms enriched by GSEA and LRpath for the comparisons among three DEG selection criteria based on the microarray data from three commercial platforms at the same test site. For test site 1, Figures 6 and 7 showed the number of GO terms enriched by GSEA and LRpath, respectively, and the percentages of overlapping GO terms for the comparisons among three microarray platforms, when the inputted DEG lists were generated by fold change ranking (|log_2_FC| > 1 (p < 0.05)), SAM (p < 0.05) and *t*-test (p < 0.05). For the GO terms enriched by GSEA, it can be seen from Figure 6 that all the percentages of overlapping GO terms for the cross-platform comparisons were around 80%, which indicated that there was no significant impact on the concordance of GO terms when the inputted DEG lists were generated by different DEG selection methods and from different microarray platforms. However, for the comparisons of AFX versus AG1 and AFX versus ILM, the percentages of GO terms enriched by LRpath were ~31% lower than those enriched by GSEA (a drop from ~83% to ~52%) when inputted DEG lists were generated by *t*-test (p < 0.05) (Figure 7). As to the comparison of AG1 versus ILM, there was also a decrease in percentage of overlapping GO terms by approximately 13% when generating DEG lists by *t*-test (p < 0.05). Note that, to some extent the number of enriched GO terms will impact on the cross-platform concordance.

## Discussion

Reproducible enrichment results are essential for further biological interpretation of microarray data when using statistical methods for gene enrichment analysis. It was proved by MAQC project that the levels of DEG concordance in inter-laboratory and cross-platform comparisons were generally high [3,4]. For the subsequent enrichment analysis, it is important to know whether the DEG lists generated by different statistical criteria from different microarray platforms can ensure satisfied reproducibility of the enrichment results. In our current study, we systematically investigated the intra- and inter-platform concordance of GO terms enriched by GSEA and LRpath. Note that GSEA is of the 'subject-sampling' type while LRpath treats the genes as the sampling units. In this study, we only focused on the concordance of GO terms enriched by the same enrichment analysis method. The comparison of different enrichment analysis methods will be discussed in our further research.

As proposed by MAQC project that combining fold change ranking with a non-stringent p-value cut-off can provide reproducible DEG lists, the levels of concordance of enriched GO terms were still high for this straightforward combining method. In inter-site comparisons for AFX, AG1 and ILM, all the percentages of overlapping GO terms enriched by GSEA and LRpath were above ~90% and ~80%, respectively, when GO terms meeting FDR < 0.25 criterion were selected. The concordance of GSEA results were no significant difference in inter-site comparisons. But for a certain test site, the concordance of LRpath results were obviously different when the comparisons among three DEG selection criteria. For the cross-platform comparisons at each test site, the percentages were around 80% (varied from ~75% to ~84%) when the GO terms were enriched with the inputted DEGs selected by fold change ranking (|log_2_FC| > 1 (p < 0.05)). By contrast, the lack of reproducibility of enriched GO terms was found when the inputted DEGs were selected by *t*-test. Although all the percentages of overlapping GO terms enriched by GSEA in inter-site comparison were still greater than ~87%, the percentages varied from ~69% to ~74% when the inputted DEGs were generated by *t*-test and the GO terms were enriched by LRpath. Similarly, in the cross-platform comparisons for *t*-test (p < 0.05), the percentages of overlapping GO terms enriched by GSEA were ~78% and then dropped to ~50% when GO terms were enriched by LRpath. In addition, for AFX and AG1, we found that the percentages of overlapping GO terms for the comparison of fold change (|log_2_FC| > 1 (p < 0.05)) versus SAM (p < 0.05) were always higher than ~76% when comparing the different DEG selection criteria at each test site. It suggested that the concordance of enrichment results based on the DEG selection methods of fold change ranking and SAM were generally high.

To some extent, the number of selected DEGs impacted on the percentages of overlapping GO terms. In inter-site comparisons, most of the percentages for SAM (p < 0.01) were higher than ~85% except for the comparisons of ILM_1 versus ILM_2 and ILM_2 versus ILM_3, when the GO terms were enriched by GSEA (Additional file 1: Figure S2a and S2c), because the number of DEGs selected from test site 2 was 3,059, which was about half of those selected from test sites 1 and 3 (Table 1). It is worthwhile to note that there were large discrepancies between the two reference RNA samples, namely UHRR and HBRR, which were just designed for investigating the capabilities and limitations of the microarray technology and for the corresponding data analysis approaches. So, the number of selected DEGs from a relevant biological study such as control versus treatment would be less than those selected by using UHRR versus HBRR, for which it may cause a decrease in the percentages of overlapping GO terms. In addition, when comparing the GO semantic similarity with real biological data sets, the hierarchical structure of GO graph should be considered [29].

## Conclusions

In our study, we conducted the intra- and inter-platform comparisons with MAQC data sets and inspected the concordance of GO terms enriched by GSEA and LRpath when the inputted DEG lists were generated by different statistical criteria. The percentages of overlapping GO terms for fold change ranking (|log_2_FC| > 1 (p < 0.05)) were as high as ~90% in inter-site comparisons when GO terms meeting FDR < 0.25 criterion were selected, and were around 80% in cross-platform comparisons. Our results demonstrated that the DEG lists generated by a straightforward method combining fold change ranking with a non-stringent p-value cut-off can ensure the reproducibility of the enrichment results. In addition, the tool GSEA for enrichment analysis can always yield relatively stable enrichment results.

## Methods

### Data sets

The MAQC data sets were downloaded from the National Center for Biotechnology Information’s Gene Expression Omnibus (GEO series accession number: GSE5350). The two compared RNA samples were a Universal Human Reference RNA (UHRR, marked as sample A) from Stratagene and a Human Brain Reference RNA (HBRR, marked as sample B) from Ambion, which were used as two compared biological conditions for selecting DEGs. Microarray data generated from three commercial platforms: Affymetrix (AFX), Agilent Technologies (AG1) and Illumina (ILM), were collected from MAQC data sets and used in our study. Each microarray platform was tested at three independent test sites and each RNA sample was replicated five times at each test site. Due to the distinct probe-design strategies and manufacturing processes, different microarray platforms target different subsets of the whole human transcriptome. For the convenient of intra- and inter-platform comparison, we directly focused on the expression of 12,091 common probes, which were summarized by MAQC project and represented 12,091 unique Entrez genes [3,4]. Results showed below were based on these 12,091 “common” genes. All the gene expression data were log_2_-transformed.

### Student *t*-test

Student *t*-test is extensively used in gene expression analysis. It demonstrates whether the difference between two groups of samples is significant. In our study, the p-values calculated by *t*-test are directly used for gene filtering without any multiple-testing correction. The DEGs were obtained by setting two criteria of p < 0.05 and p < 0.01, and inputted to GSEA and LRpath for further analysis.

### Fold Change (FC)

The fold change is a wildly used method for selecting DEGs from gene expression data and indicates to what extent a gene is differentially expressed between two groups of samples. After filtering the genes with the non-stringent p-value cutoff (p < 0.05 or p < 0.01) which calculated by *t*-test, the rest of them were ranked by their fold changes (sample A/sample B). Note that for each test site, the expression intensity of a gene in sample A or sample B was the average value of the intensities of five replicates. Then, at each given cut-off, a list of DEGs was obtained for the subsequent analysis.

### Significance Analysis of Microarrays (SAM)

Significance analysis of microarrays (SAM) identifies whether a gene is significantly different between two groups of samples based on a permutation procedure by combining the gene-specific *t*-test with a statistic *d* value [28]. DEGs selected by SAM were calculated with siggenes package in Bioconductor 2.10 within R 2.12.1.

### Gene Set Enrichment Analysis (GSEA)

Gene Set Enrichment Analysis (GSEA) is a commonly used approach for enrichment analysis. An earlier version of this method was firstly proposed by Mootha et al. [9]. In 2005, Subramanian et al. extended this procedure by considering the correlation between each of the genes and the phenotypes [10]. In our research, we used the GSEA methodology described by Subramanian et al. GSEA software can be downloaded from web site (http://www.broadinstitute.org/gsea/index.jsp). In the calculation procedure, genes were first ranked by signal to noise ratio (SNR) or other metric generated by statistical methods, such as p-values generated by standard *t*-test and SAM or log_2_-transformed values of fold change. Then an enrichment score (ES) corresponded to Kolmogorov-Smirnow statistic was calculated based on the ranked gene lists for each predefined gene set and subsequently normalized according to its size. Finally, based on the normalized enrichment score, a permutation-based false discovery rate (FDR) was generated to indicate the significance of enriched gene sets. The GO terms associated with the significant enriched gene sets were identified and used for further biological interpretation.

### LRpath

LRpath is a logistic-based method for identifying the significantly enriched gene sets, which described the log-odds of a gene belonging to the specific category as a linear function of the statistical significance of its expression level, e.g. p-value generated by *t*-test [22]. The slop parameter in the logistic regression equation was used to decide whether a predefined gene set is significantly enriched with the inputted DEGs. The p-values from the test of each predefined gene sets were then adjusted for multiple testing by controlling FDR. LRpath program was run in R 2.12.1 and can be downloaded from web site (http://eh3.uc.edu/lrpath).

### Percentage of overlapping GO terms

The percentage of overlapping GO terms is a measure of the concordance of significant GO terms discovered by enrichment analysis. Only the GO terms with FDR < 0.25 criterion were considered as significant and ranked by FDR in ascending order. The two lists of significant GO terms are compared under the same length, which was decided by the shorter one. The percentage of overlapping GO terms was calculated as follow:

$$\text{Percentage}\;\text{of}\;\text{overlapping}\;\text{GO}\;\text{terms}=\frac{O_{i}}{T_{i}}\times 100\%$$

where *O*_*i*_ is the number of pairs of overlapped GO terms and *T*_*i*_ is the total number of pairs of two lists within the first *i* pairs (i = 1, 2,…, N). For GSEA, N is the length of combined list which consisted of the shorter lists of GO terms between two compared lists enriched in ‘pos’ and ‘neg’ phenotypes. For LRpath, N equals the shorter length of two compared lists.

## Abbreviations

SAM: Significance analysis of microarrays; FDR: False discovery rate; DEGs: Differentially expressed genes; GO: Gene Ontology; GSEA: Gene set enrichment analysis.

## Competing interests

The authors declare that they have no competing interests.

## Authors’ contributions

ZW, LZ conceived the study design, carried out the data analysis and drafted the manuscript. JZ, GY, DW, LJ helped in analysis and discussion, ML refined the manuscript and gave useful comments. ML, ZW initialized and supervised the whole project. All authors read and approved the final manuscript.

## Supplementary Material

Additional file 1 **A PDF file containing the supplemental figures.** It includes figures of intra-platform concordance of significant GO terms and figures of the concordance of significant GO terms within the same test site and among three DEGs selected methods. Click here for file
