# Supplementary material for: Investigating the concordance of Gene Ontology terms reveals the intra- and inter-platform reproducibility of enrichment analysis
Source: BMC Bioinformatics. 2013 Apr 29;14:143. doi: 10.1186/1471-2105-14-143 (PMC3644270; doi:10.1186/1471-2105-14-143)
Supplement: Additional file 1 — A PDF file containing the supplemental figures. It includes figures of intra-platform concordance of significant GO terms and figures of the concordance of significant GO terms within the same test site and among three DEGs selected methods. [file 1471-2105-14-143-S1.pdf]

Investigating the concordance of Gene Ontology terms reveals the intra- and inter-platform reproducibility of enrichment analysis

Lifang Zhang[1], Juan Zhang[1], Gang Yang[1], Di Wu[1], Lina Jiang[1], Zhining Wen[1,2]*, Menglong Li[1]*

Supplementary materials

Figure S1	Intra-platform concordance of significant GO terms enriched by GSEA among three test sites of AG1
Figure S2	Intra-platform concordance of significant GO terms enriched by LRpath among three test sites of AG1
Figure S3	Intra-platform concordance of significant GO terms enriched by GSEA among three test sites of ILM
Figure S4	Intra-platform concordance of significant GO terms enriched by LRpath among three test sites of ILM
Figure S5	The concordance of significant GO terms enriched by GSEA within the same test site of AG1 and among three DEGs selected methods
Figure S6	The concordance of significant GO terms enriched by LRpath within the same test site of AG1 and among three DEGs selected methods
Figure S7	The concordance of significant GO terms enriched by GSEA within the same test site of ILM and among three DEGs selected methods
Figure S8	The concordance of significant GO terms enriched by LRpath within the same test site of ILM and among three DEGs selected methods

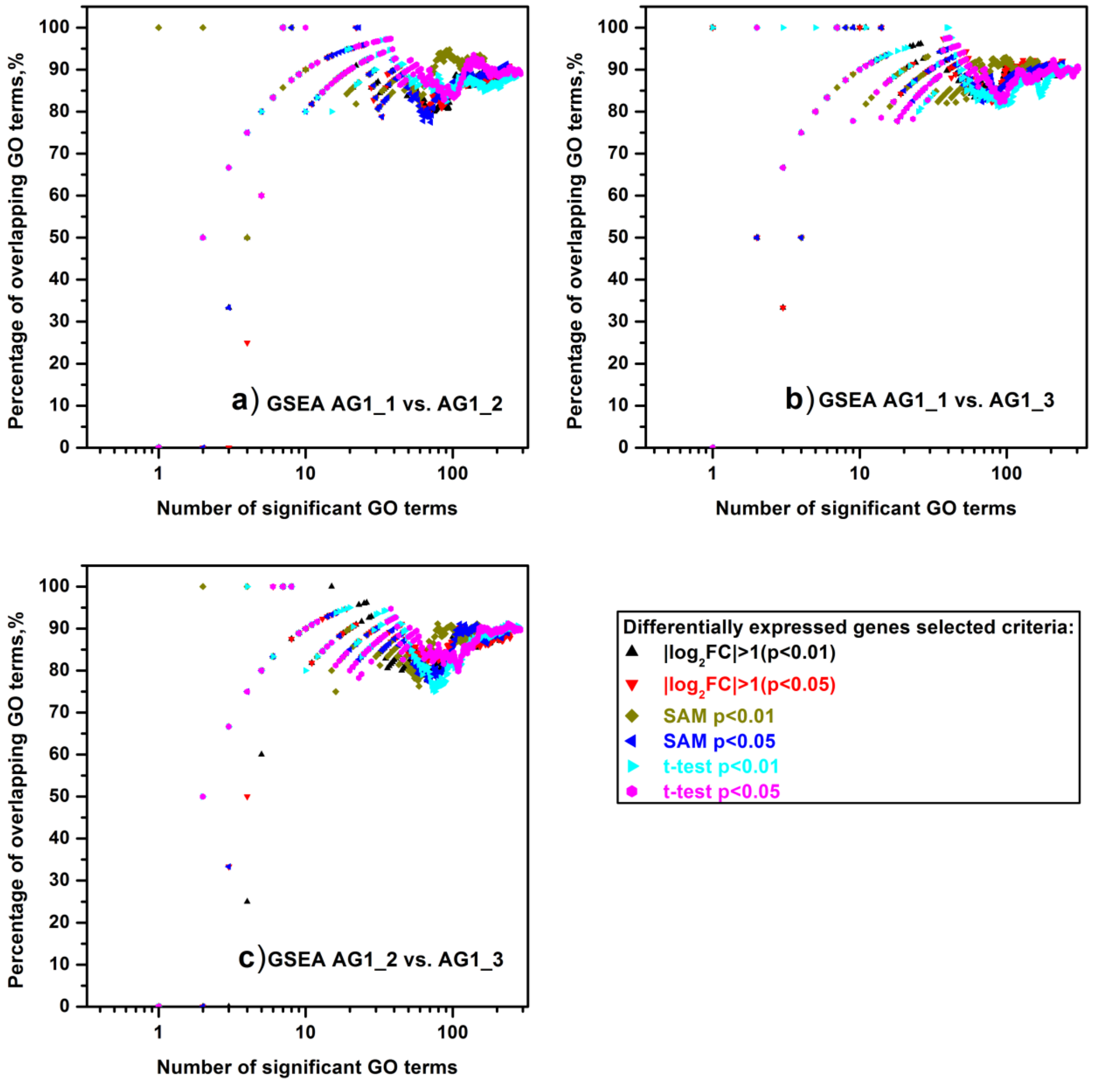

Figure S1 - Intra-platform concordance of significant GO terms enriched by GSEA among three test sites of AG1. (a) AG1_1 versus AG1_2; (b) AG1_1 versus AG1_3; (c) AG1_2 versus AG1_3. The scatter plots showed the percentages of overlapping GO terms which were enriched by GSEA and derived from two test sites. Each color and each type of points represented the different gene lists that selected by different statistical methods and different cutoff. The x-axis represents the number of enriched GO terms selected as significance, and y-axis is the percentage (%) of GO terms common to the two AG1 test sites enrichment results. The _1, _2 and _3 suffixes refer to test sites.

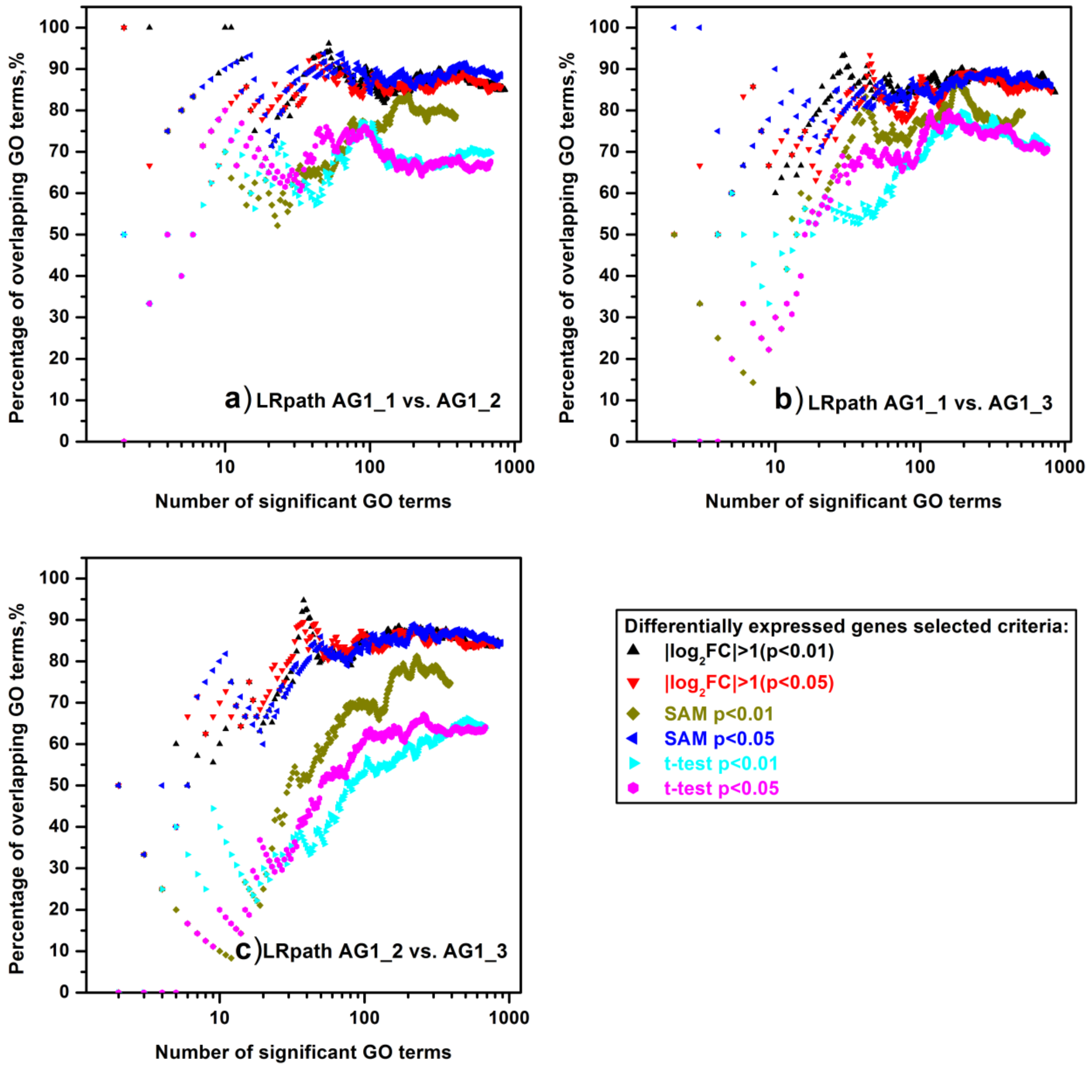

Figure S2 - Intra-platform concordance of significant GO terms enriched by LRpath among three test sites of AG1. (a) AG1_1 versus AG1_2; (b) AG1_1 versus AG1_3; (c) AG1_2 versus AG1_3. The scatter plots showed the percentages of overlapping GO terms which were enriched by LRpath and derived from two test sites. See notes under figure S1 for more information.

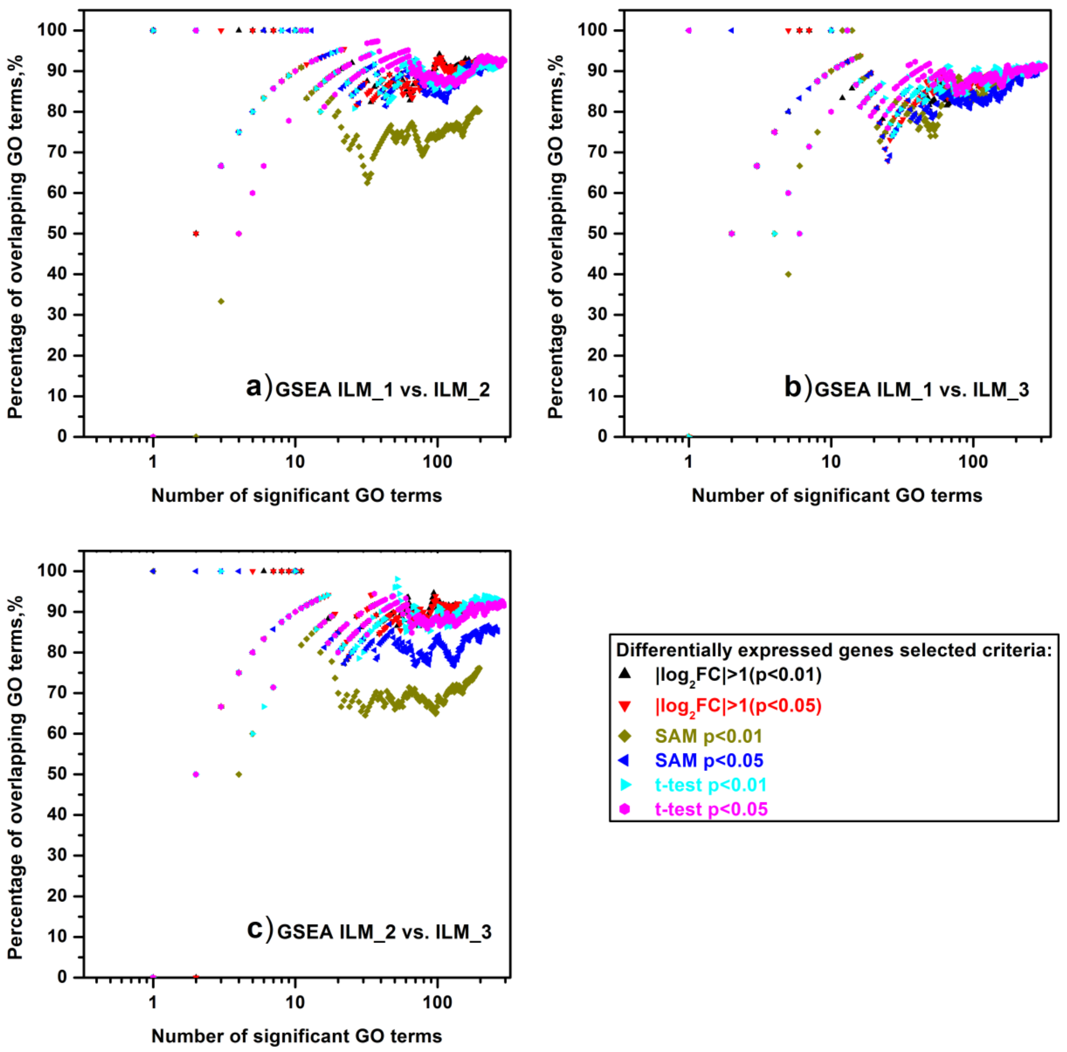

Figure S3 - Intra-platform concordance of significant GO terms enriched by GSEA among three test sites of ILM. (a) ILM_1 versus ILM_2; (b) ILM_1 versus ILM_3; (c) ILM_2 versus ILM_3. The scatter plots showed the percentage of overlaps GO terms which were enriched by GSEA and derived from two test sites. See notes under figure S1 for more information.

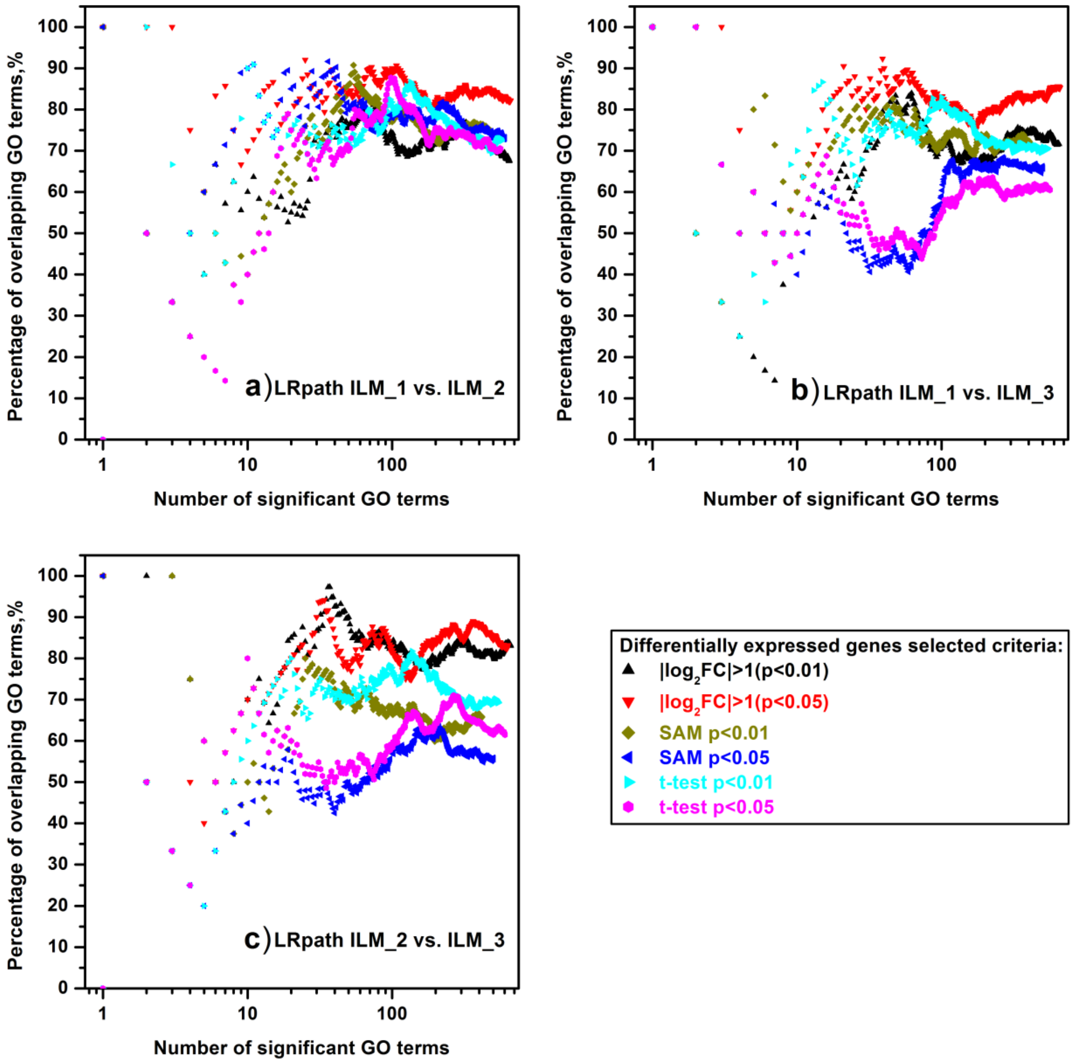

Figure S4 - Intra-platform concordance of significant GO terms enriched by LRpath among three test sites of ILM. (a) ILM_1 versus ILM_2; (b) ILM_1 versus ILM_3; (c) ILM_2 versus ILM_3. The scatter plots showed the percentage of overlaps GO terms which were enriched by LRpath and derived from two test sites. See notes under figure S1 for more information.

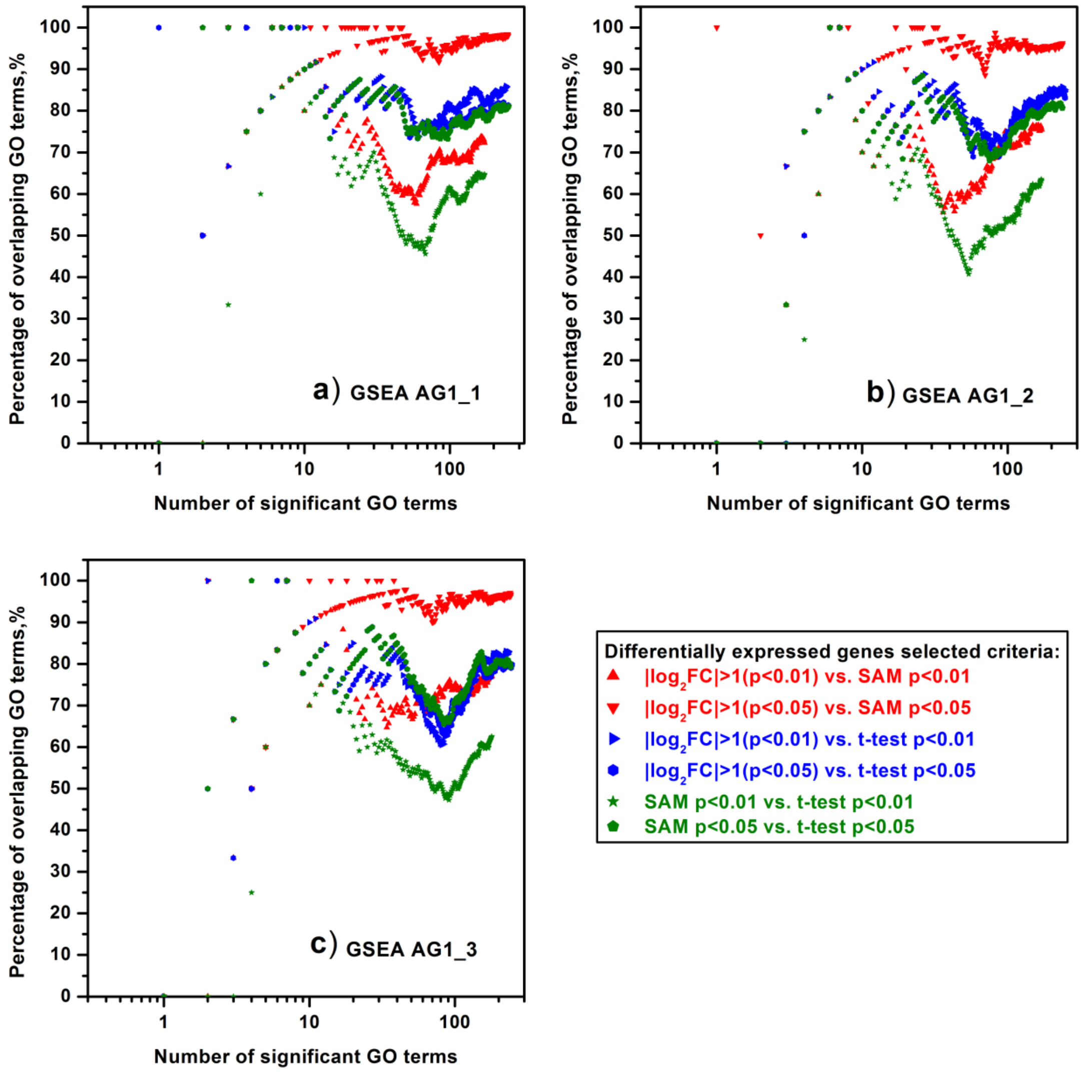

Figure S5 - The concordance of significant GO terms enriched by GSEA within the same test site of AG1 and among three DEGs selected methods. Four DEG lists generated by fold change ranking, two DEG lists generated by SAM and two DEG lists generated by t-test were inputted in GSEA to enrich significant GO terms (FDR<0.25). And then the concordance of two significant GO terms lists derived from two DEGs selected methods was compared on the same p-value cutoff. The red markers reflect the percentages of overlapping GO terms by using fold change ranking and SAM for generating DEG lists. The blue markers reflect the percentages of overlapping GO terms by using fold change ranking and t-test for generating DEG lists. The olive markers reflect the percentages of overlapping GO terms by using SAM and t-test for generating DEG lists. The _1, _2 and _3 suffixes refer to test site location.

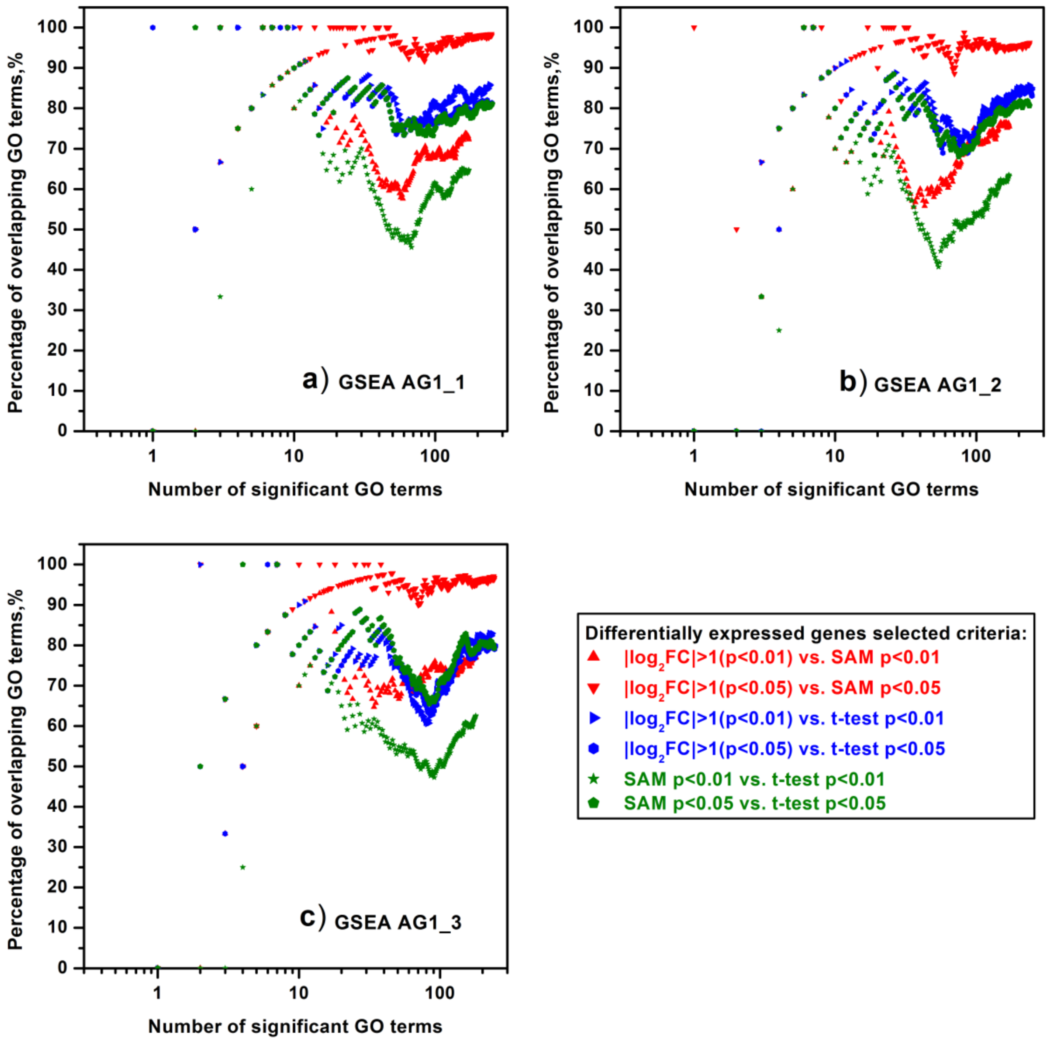

Figure S6 - The concordance of significant GO terms enriched by LRpath within the same test site of AG1 and among three DEGs selected methods. Comparisons of significant GO terms enriched by LRpath were conducted among three DEGs selection methods within the same test site of AG1. See notes under figure S5 for more information.

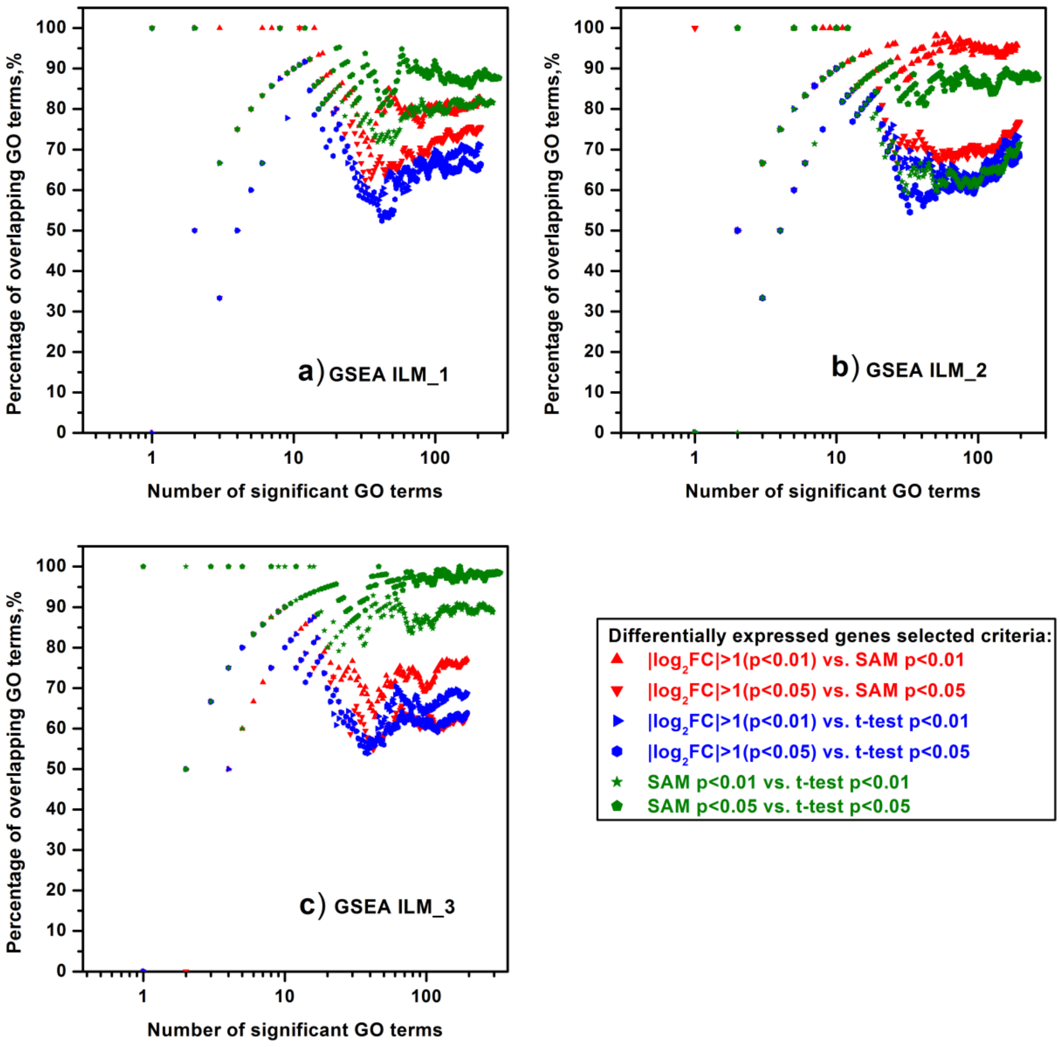

Figure S7 - The concordance of significant GO terms enriched by GSEA within the same test site of ILM and among three DEGs selected methods. Comparisons of significant GO terms enriched by GSEA were conducted among three DEGs selection methods within the same test site of ILM. See notes under figure S5 for more information.

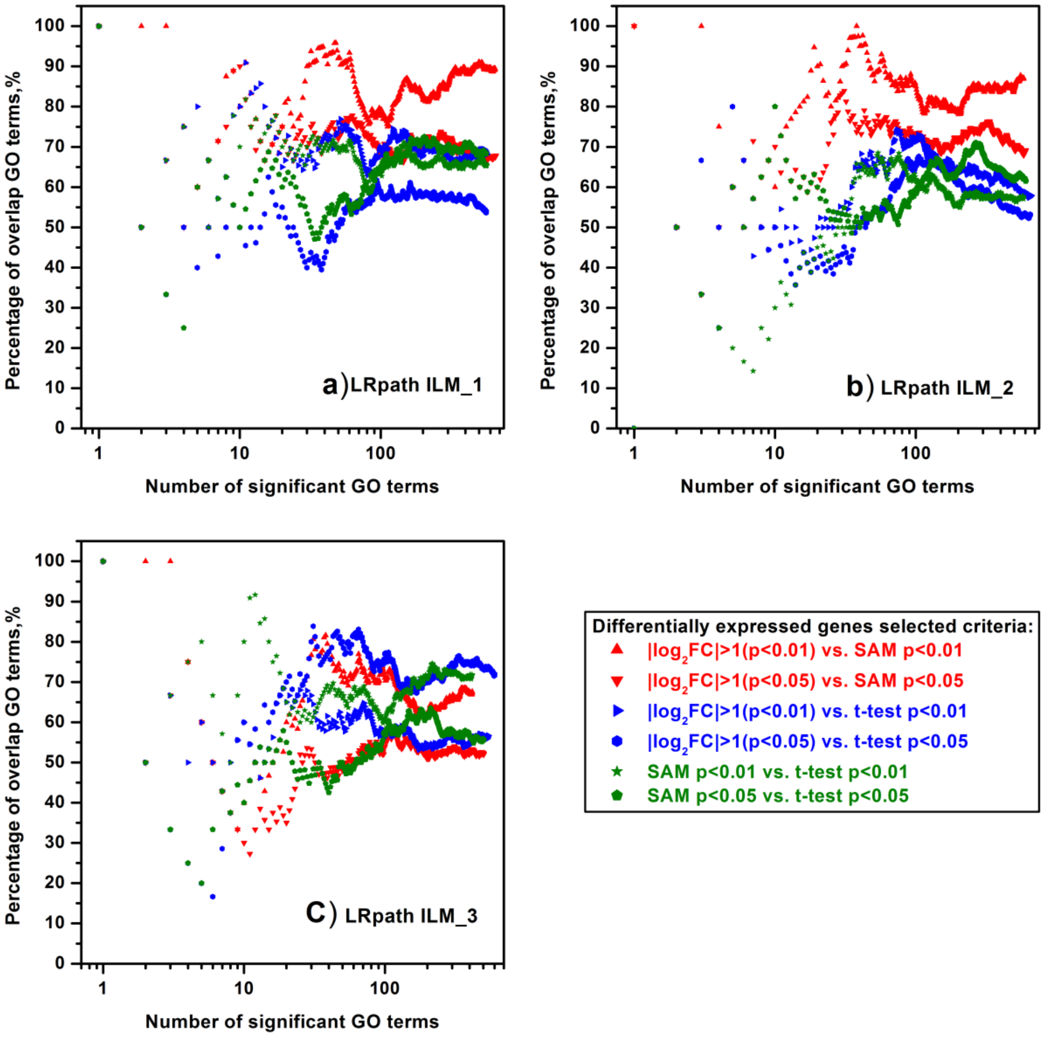

Figure S8 - The concordance of significant GO terms enriched by LRpath within the same test site of ILM and among three DEGs selected methods. Comparisons of significant GO terms enriched by LRpath were conducted among three DEGs selection methods within the same test site of ILM. See notes under figure S5 for more information.
